# Supplementary material for: TogoMCP: natural language querying of life-science knowledge graphs via schema-guided LLMs and the Model Context Protocol
Source: Database (Oxford). 2026 Jul 24;2026:baag042. doi: 10.1093/database/baag042 (PMC13397537; doi:10.1093/database/baag042)
Supplement: baag042_Supplemental_Files [file baag042_supplemental_files.zip › supplementary_table_S1.pdf]

Supplementary Table S1

Per-question score deltas across ablation conditions
TogoMCP: Natural Language Querying of Life-Science Knowledge Graphs via Schema-Guided LLMs and the Model Context Protocol

This table accompanies the main manuscript (section "Ablation Study: Contributions of MIE Files and the Usage Guide", Table 2). All scores are means over five evaluation runs using an LLM judge (Claude Opus 4.7) on a 4–20 scale. Δ values are TogoMCP score minus baseline score for each ablation condition. Bold Δ values indicate gains ≥2.0; red values indicate negative deltas (TogoMCP underperformed baseline). Conditions: WG = With Guide (full system); MIE-Instr (no Usage Guide, explicit MIE instruction); No-Instr (no Usage Guide, no instruction); No MIE (`get_MIE_file` excluded).

Table 1: Per-question score deltas across all four ablation conditions. Scores are averages over five evaluation runs (LLM judge, 4–20 scale). Δ values are TogoMCP minus baseline for each condition. Type abbreviations: Y/N = yes/no, Fact = factoid, List = list, Sum = summary, Cho = choice.

**Q**	**Type**	**Baseline**	WG	ΔWG	ΔMIE-Instr	ΔNo-Instr	ΔNo MIE
Q001	Y/N	18.0	20.0	**+2.0**	+1.6	**+2.2**	+0.8
Q007	Y/N	13.4	19.8	**+6.4**	**+7.6**	**+7.8**	**+6.0**
Q012	Y/N	15.0	19.4	**+4.4**	**+6.4**	**+3.0**	**+3.6**
Q017	Y/N	16.8	19.8	**+3.0**	**+2.4**	-3.2	-2.4
Q020	Y/N	13.8	20.0	**+6.2**	**+6.6**	**+6.8**	**+6.6**
Q026	Y/N	17.0	20.0	**+3.0**	**+2.8**	**+2.4**	**+2.2**
Q032	Y/N	17.2	20.0	**+2.8**	**+3.4**	+0.6	+0.2
Q036	Y/N	14.0	20.0	**+6.0**	**+6.4**	**+6.6**	**+5.6**
Q042	Y/N	16.4	19.4	**+3.0**	**+3.2**	**+2.0**	**+4.0**
Q046	Y/N	14.6	20.0	**+5.4**	**+6.0**	**+5.0**	**+3.4**
Q002	Fact	14.0	17.8	**+3.8**	**+3.2**	**+3.0**	**+4.4**
Q003	Fact	13.8	16.8	**+3.0**	**+5.4**	**+7.2**	**+2.6**
Q006	Fact	13.8	16.8	**+3.0**	+1.4	**+3.4**	**+3.4**
Q011	Fact	13.8	16.8	**+3.0**	**+2.0**	**+5.2**	**+4.8**
Q014	Fact	13.8	19.6	**+5.8**	**+5.4**	**+7.0**	**+6.6**
Q022	Fact	13.8	16.8	**+3.0**	**+2.8**	**+4.6**	**+4.4**
Q027	Fact	13.8	15.8	**+2.0**	+1.4	**+2.6**	**+5.0**
Q031	Fact	13.8	16.0	**+2.2**	**+4.2**	**+2.6**	**+2.8**
Q043	Fact	13.8	20.0	**+6.2**	**+5.8**	**+6.8**	**+6.6**
Q047	Fact	13.8	20.0	**+6.2**	**+5.8**	**+3.0**	**+2.8**
Q009	List	15.0	16.2	+1.2	**+2.0**	+0.4	**+2.8**
Q013	List	16.2	18.6	**+2.4**	-1.2	+1.8	**+3.2**
Q018	List	14.2	19.2	**+5.0**	**+6.2**	**+5.8**	**+5.2**
Q023	List	14.0	17.6	**+3.6**	**+3.6**	**+6.2**	**+2.6**
Q028	List	14.0	20.0	**+6.0**	**+6.2**	**+6.2**	**+6.8**
Q033	List	14.0	17.6	**+3.6**	**+3.2**	**+6.4**	**+5.4**
Q039	List	14.0	20.0	**+6.0**	**+5.8**	**+6.2**	**+3.0**
Q040	List	15.0	18.8	**+3.8**	**+2.0**	**+5.2**	**+3.6**
Q044	List	12.8	12.8	+0.0	**+2.6**	+0.6	**+5.0**
Q048	List	13.8	18.0	**+4.2**	**+3.6**	**+4.0**	**+2.2**
Q004	Sum	16.0	17.0	+1.0	+1.8	+1.4	+0.0
Q008	Sum	15.2	17.2	**+2.0**	**+4.4**	+0.0	+0.4
Q015	Sum	15.2	16.8	+1.6	**+4.0**	-0.8	+1.2
Q021	Sum	15.0	14.8	-0.2	**+2.6**	**+2.0**	**+3.0**

continued on next page

Table 1 – *continued*

**Q**	**Type**	**Baseline**	WG	ΔWG	ΔMIE-Instr	ΔNo-Instr	ΔNo MIE
Q024	Sum	16.4	19.6	**+3.2**	+1.2	**+3.8**	**+3.2**
Q029	Sum	14.8	20.0	**+5.2**	**+4.4**	**+3.4**	**+3.2**
Q034	Sum	14.8	18.0	**+3.2**	**+4.4**	+0.0	-0.2
Q037	Sum	14.8	18.2	**+3.4**	**+3.2**	-1.0	**+2.2**
Q045	Sum	17.2	18.4	+1.2	+1.2	+0.4	+1.2
Q049	Sum	15.8	19.6	**+3.8**	**+3.6**	**+3.4**	**+3.2**
Q005	Cho	18.0	20.0	**+2.0**	+0.0	+1.0	+0.2
Q010	Cho	14.2	20.0	**+5.8**	**+7.2**	**+6.8**	**+5.0**
Q016	Cho	17.6	18.6	+1.0	+0.4	**+6.6**	+0.6
Q019	Cho	13.8	20.0	**+6.2**	**+6.0**	+0.2	+1.0
Q025	Cho	18.0	19.0	+1.0	+1.6	+1.0	+1.2
Q030	Cho	17.0	18.2	+1.2	-1.0	+0.0	+1.0
Q035	Cho	17.8	19.0	+1.2	+0.8	+1.6	+0.2
Q038	Cho	14.0	20.0	**+6.0**	+1.6	+1.4	+1.2
Q041	Cho	17.8	19.4	+1.6	+1.2	+1.4	+1.2
Q050	Cho	14.0	20.0	**+6.0**	**+6.4**	**+7.0**	**+5.6**
**Mean**		**15.10**	**18.55**	**+3.45**	**+3.46**	**+3.22**	**+2.96**
