# Supplementary material for: TogoMCP: natural language querying of life-science knowledge graphs via schema-guided LLMs and the Model Context Protocol
Source: Database (Oxford). 2026 Jul 24;2026:baag042. doi: 10.1093/database/baag042 (PMC13397537; doi:10.1093/database/baag042)
Supplement: baag042_Supplemental_Files [file baag042_supplemental_files.zip › supplementary_table_S2.pdf]

Supplementary Table S2

Aggregate distribution and pairwise statistical tests of ablation conditions
TogoMCP: Natural Language Querying of Life-Science Knowledge Graphs via Schema-Guided LLMs and the Model Context Protocol

This table accompanies the main manuscript (section "Ablation Study: Contributions of MIE Files and the Usage Guide", sub-paragraphs "All conditions outperform baseline; MIE files are the primary contributor to the mean", "The MIE instruction captures the full benefit of the Usage Guide *on average*", and "Component value hierarchy and the Usage Guide's worst-case role"). All scores are means over five evaluation runs using an LLM judge (Claude Opus 4.7) on a 4–20 scale. Per-question Δ is the TogoMCP score minus baseline score, computed within each condition's matched no-tool run.

Conditions: WG = With Guide (full system); MIE-Instr = no Usage Guide, but explicit instruction to call `find_databases` and `get_MIE_file`; No-Instr = no Usage Guide, no instructions; No MIE = `get_MIE_file` excluded entirely.

Panel A. Distribution of per-question mean Δ (n = 50 per condition).

Condition	min	Q_{10}	Q_{25}	median	mean	Q_{75}	Q_{90}	max	SD
WG	−0.20	+1.18	+2.00	+3.10	+3.45	+5.35	+6.02	+6.40	1.90
MIE-Instr	−1.20	+1.16	+1.65	+3.20	+3.46	+5.70	+6.40	+7.60	2.21
No-Instr	−3.20	+0.00	+1.10	+3.00	+3.22	+6.10	+6.80	+7.80	2.71
No MIE	−2.40	+0.20	+1.20	+3.00	+2.96	+4.70	+5.64	+6.80	2.14

Means match the corresponding row of Table 2 in the main manuscript (within rounding). Between-question SD is computed across the 50 per-question mean Δ values for each condition.

Panel B. Pairwise statistical tests, With Guide as reference.

Comparison vs. WG (other condition)	Mean Δ gap (WG − other)	Wilcoxon p (two-sided)	Fisher loss p (one-sided)	Levene SD p (WG vs. other)
MIE-Instr	−0.004	0.877	0.036	0.265
No-Instr	+0.232	0.739	0.009	0.005
No MIE	+0.496	0.067	0.087	0.565

Mean Δ gap: difference in mean per-question Δ between With Guide and the comparison condition; positive values indicate that With Guide produced a larger improvement on average. *Wilcoxon p*: paired Wilcoxon signed-rank test on the 50 per-question mean Δ values (two-sided). *Fisher loss p*: Fisher's exact one-sided test on the 2×2 contingency of loss (TogoMCP score below baseline) vs. no-loss evaluations across the 250 question–run pairs per condition; tests whether With Guide produces fewer losses than the comparison condition. *Levene SD p*: Levene test for equality of between-question SD (50 per-question mean Δ values) under center=mean. Worst-case Wilcoxon p values (paired comparison of per-question worst-case TogoMCP score across five evaluator runs) are reported inline in the main manuscript and are not repeated here.

Panel B (continued). Bootstrap confidence intervals for the paired difference (WG − other), n = 20,000 resamples of the 50 per-question paired differences.

Comparison vs. WG	95% CI	90% CI
MIE-Instr	[−0.40, +0.41]	[−0.34, +0.34]
No-Instr	[−0.38, +0.85]	[−0.28, +0.75]
No MIE	[−0.06, +1.04]	[+0.04, +0.94]

The 95% CI for WG − No MIE just barely includes zero ([−0.06, +1.04]), which is consistent with the marginally non-significant two-sided Wilcoxon $p = 0.067$ in Panel B. The 90% CI for the same comparison excludes zero ([+0.04, +0.94]), indicating that under a one-sided directional hypothesis (removing MIE files does not improve performance) the WG > No MIE direction is supported. The Cohen's d on the per-question paired difference for the WG vs. No MIE comparison is 0.25.

Panel C. Per-condition loss summary.

Condition	Eval. loss rate (out of 250)	Q net-loss (out of 50)	min Δ (per-q mean)	mean 5-run SD (TogoMCP)	mean worst (min-of-5)	Net-loss question IDs (Δ)
WG	1.6% (4/250)	1	−0.20	0.464	18.02	q021 (−0.20)
MIE-Instr	4.8% (12/250)	2	−1.20	0.580	17.84	q013 (−1.20), q030 (−1.00)
No-Instr	6.0% (15/250)	3	−3.20	0.660	17.24	q017 (−3.20), q037 (−1.00), q015 (−0.80)
No MIE	4.0% (10/250)	2	−2.40	0.518	17.56	q017 (−2.40), q034 (−0.20)

Eval. loss rate: fraction of 250 individual question–run evaluations where the TogoMCP score fell below the matched baseline score. *Q net-loss*: number of distinct questions (out of 50) whose 5-run mean Δ was negative. *min* Δ: lowest per-question mean Δ across the 50 questions. *mean 5-run SD*: average over 50 questions of the within-question SD of the five TogoMCP total-score evaluations. *mean worst (min-of-5)*: average over 50 questions of the minimum TogoMCP total across the five evaluator runs; this is the statistic used in the worst-case paired Wilcoxon comparison reported in the main manuscript.
